# Comparison of whole trunk muscle mass between healthy and lumbar herniated nucleus pulposus patients using abdominal pelvic computed tomography

**DOI:** 10.3389/fmed.2023.1190021

**Published:** 2023-08-01

**Authors:** Chi-Hoon Oh, Dong-Eun Shin, Siyeong Yoon, Jongbeom Oh, Younghun Lee, Soonchul Lee

**Affiliations:** Department of Orthopaedic Surgery, CHA Bundang Medical Center, CHA University School of Medicine, Seongnam-si, Gyeonggi-do, Republic of Korea

**Keywords:** herniated nucleus pulposus, trunk skeletal muscle imbalance, sarcopenia, abdominal pelvic computed tomography, paraspinal muscle

## Abstract

**Background:**

Paraspinal muscle asymmetry is associated with low back pain (LBP) problems. This study aimed to analyze skeletal muscle areas around the lumbar vertebra and compare the ratio of anterior and posterior muscles between patients with lumbar herniated nucleus pulposus (HNP) and healthy individuals using abdominal pelvic computed tomography (APCT).

**Methods:**

After propensity score matching, 122 HNP patients and 122 non-HNP individuals were finally used for analyses. In APCT, axial cut images were collected at the level of the third lumbar vertebra lower end plate and only the muscle structure was obtained. After obtaining the muscular portion of their image, we measured the skeletal muscle area (SMA). Second, for analysis relation of sarcopenia and HNP, the status of low skeletal muscle mass was determined using a previously reported criteria based on APCT scans in the Republic of Korea.

**Results:**

From the analysis of the anterior–posterior muscles, the ratio of anterior SMA was statistically significantly larger in the HNP group than in the non-HNP group. Regarding the anatomical classification of trunk muscles, a statistically significant left–right imbalance to peripheral muscle in HNP men was observed. Regarding the status of low skeletal muscle mass, no statistical difference in prevalence between the two groups were observed. Moreover, no statistical difference in the prevalence of low skeletal muscle mass obesity was observed.

**Conclusion:**

The lumbar flexor muscle was larger in HNP than others, showing trunk muscle imbalance. However, low skeletal muscle mass is not associated with HNP.

## 1. Introduction

Low back pain (LBP) is a common symptom experienced by people of all ages (1, 2). The percentage of people who have LBP at any given time varies from 9.4% to 28.4%, while more than 80% are likely to experience LBP in their lifetime (3, 4). Herniation of the nucleus pulposus (HNP) is one of the important causes of low back pain. Internal disc disruption of the lumbar intervertebral discs is thought to be responsible for between 26% and 42% of chronic low back pain (5).

On the other hand, recent studies show that sarcopenia tends to increase with age, causes low back pain, and deteriorates the quality of life (6–8). In addition, sarcopenia of the lumbar paraspinal muscles has been receiving renewed attention as a cause of spinal degeneration (9). Both atrophy and fatty changes in paraspinal muscles originating from sarcopenia are associated with functional disorders and chronic LBP (10).

Evidence suggests that atrophy and degenerative changes in the low back muscles are associated with chronic LBP (11, 12). Moreover, studies have demonstrated that paraspinal muscle asymmetry with fatty infiltration is associated with LBP problems (11, 13, 14). Although, more asymmetry and fatty infiltration are expected to occur as the duration of symptoms increases (15), rapid morphological changes of the multifidus muscle have been observed following nerve root injury (16). A previous study reported that the skeletal muscle area (SMA) of multifidus muscle was reduced by disc herniation when symptom duration was 3 months or more (17). However, these previous studies mainly analyzed the spinal muscle through magnetic resonance imaging.

To our best knowledge, no analysis related to all trunk muscles, including abdominal muscles, has been conducted previously. Although, a few studies have examined the degree of multifidus asymmetry and fatty infiltration in patients with acute and subacute symptomatic HNP (17, 18). Based on the existing literature, it is recognized HNP can be considered one of the factors contributing to low back pain. As mentioned earlier, recent studies have also reported an association between sarcopenia and low back pain. This study focused on examining the impact of HNP on skeletal muscles and whether it contributes to low back pain, distinct from the low back pain resulting from age-related decline in skeletal muscle mass. The objective was to investigate the relationship between HNP, muscle atrophy and imbalance in patients with HNP patient compared to healthy controls (non-HNP). Hence, we measured the whole trunk SMA using abdomen and pelvic CT (APCT) after propensity score matching between HNP and non-HNP patients. Moreover, the relationship between HNP and low skeletal muscle mass closely related to sarcopenia was investigated.

## 2. Materials and methods

### 2.1. Study population

This was a retrospective study of patients who underwent at Bundang CHA Medical Center from January 2000 to April 2020. The reason for the patient’s visit to the hospital includes all routes, such as hospitalization, outpatient treatment, and visits to the emergency room. This study was approved by the CHA Bundang Medical Center Institutional Review Board (number 2021-06-037). HNP was diagnosed with typical root symptoms using magnetic resonance imaging (MRI). The level of HNP was L4–L5 or L5–S1. It was included in the patient group only when APCT was performed for trivial medical purposes (e.g., due to abdominal pain, trauma, etc.) before or after 3 months of HNP diagnosis (Supplementary Table 1). To minimize the period during which diseases diagnosed by APCT affect lumbar skeletal muscle, the period between HNP diagnosis and when APCT was performed should not to exceed several months.

**Figure 1:**
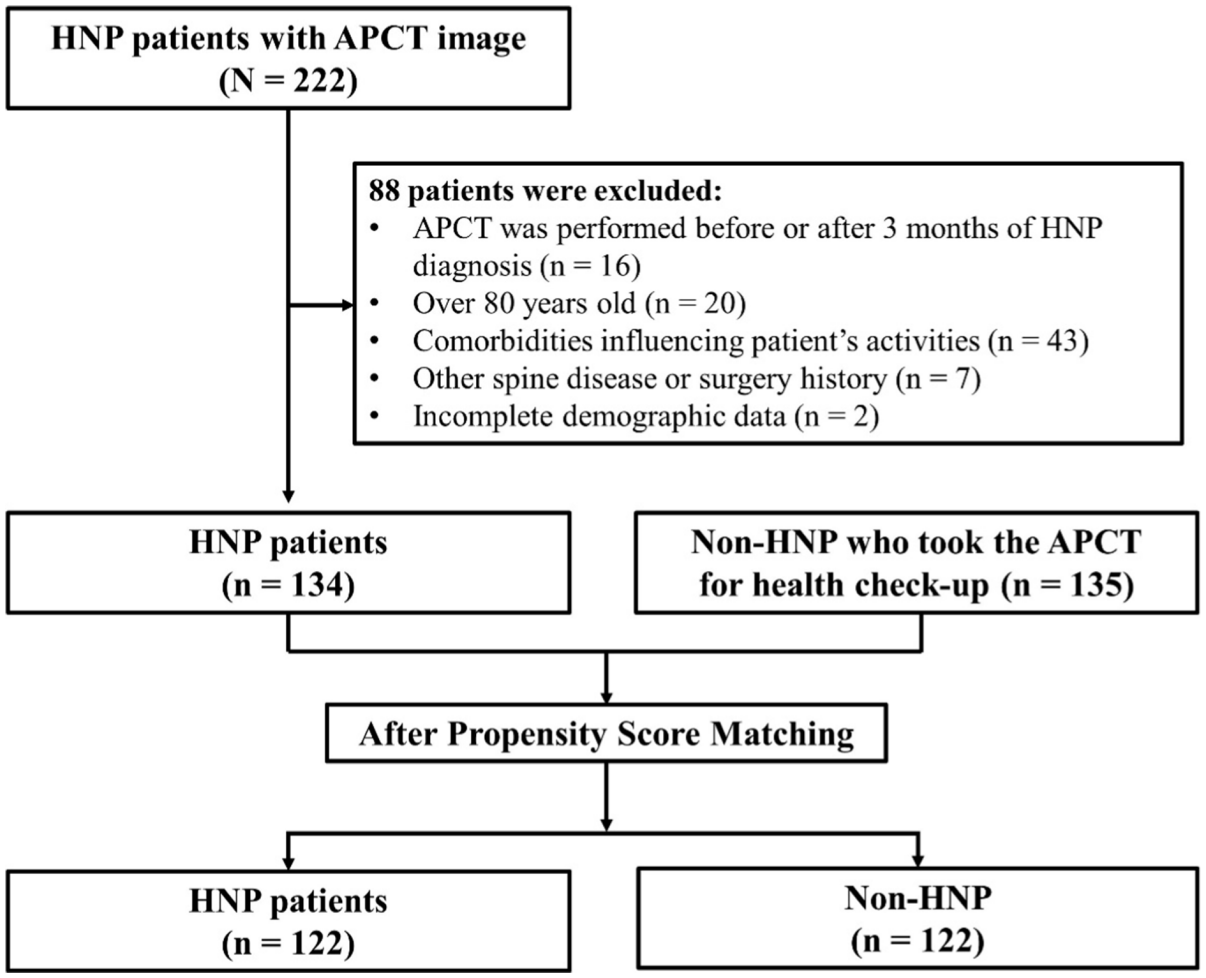
Study population. Initially, 222 HNP patients were recruited, of which 88 subjects were excluded from the study. Finally, 134 subjects were finally enrolled in this study. We applied Propensity score matching method to reduce possible bias originated from the difference in patient’s demographic characteristics. Overall, 122 patients with HNP and 122 controls were enrolled before statistical comparison. HNP, Herniated nucleus pulposus; APCT, Abdomen pelvis compute tomography.

APCT was used only to measure SMA. Initially, 222 patients were recruited, of which 88 were excluded based on the following exclusion criteria: (1) APCT was performed before or after 3 months of HNP diagnosis. (2) Over 80 years old. (3) Those with comorbidities influencing musculoskeletal activities (such as cancer and cerebrovascular accident). (4) Those with a spinal disease or fracture history except for HNP (such as scoliosis, spondylolisthesis, and compression fracture) or who had a surgery history of HNP. (5) Incomplete demographic data. As a control group, data of people who underwent APCT for health check-ups in the health promotion center without any specific history or disease, including HNP, were collected simultaneously (*N* = 135). First, demographic data, including age, sex, height, weight, and body mass index (BMI) as weight/height^2^ (kg/m^2^), were collected. Subsequently, propensity score matching (PSM) was performed. Finally, participants were divided into two groups based on whether they were diagnosed with HNP or not (*n* = 122 in each; Figure 1).

### 2.2. Measurement of muscle area

APCT examinations were performed using Revolution CT (GE Healthcare, Milwaukee, WI, United States), Lightspeed VCT (GE Healthcare, Milwaukee, WI, United States), and Optima 660(GE Healthcare, Milwaukee, WI, United States). CT examinations were performed with the following parameters: 140 kVp; automated dose modulation; Revolution CT: 256 slices and collimation of 0.625 mm, 16 mm thick coverage slice, Lightspeed VCT, Optima 660: 64 slices and collimation of 0.625 mm, 40 mm thick coverage slice. All data were reconstructed DICOM images with a 5 mm slice thickness.

We extracted an axial cut DICOM image of APCT at the level of the L3–4 intervertebral disc, which was obtained at the lower margin vertebra using a picture archiving and communicating system (PACS; Marotech Co., Ltd., Seoul, Korea). To avoid misnumbering the vertebral body by lumbosacral transitional vertebrae as much as possible, the lowest rib level was assumed to be T12 and counted in a caudal direction through the APCT test (19). To obsolete internal organs and bony structures, we drew their outlines with the region of interest (ROI) using a program (CTAn-Bruker Skyscan micro-CT). We set the lower gray threshold to 88 and the upper gray threshold to 165 when trying to extract only the muscle structure of the ROI using CTAn. After going through the despeckle process of deleting speckles generated through the automated process, the completed image was saved to check whether the muscle structure was extracted as intended.

**Figure 2:**
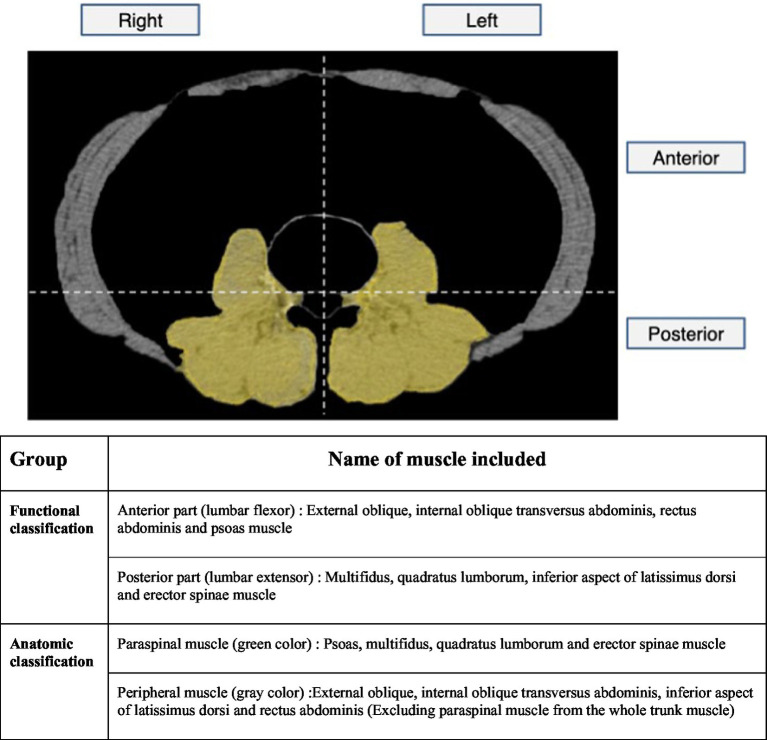
Skeletal muscle area measurement on APCT image. First, we tried to look at the relationship with HNP in terms of skeletal muscle function. The anterior muscles were composed of lumbar flexors and the posterior muscles were composed of lumbar extensors and the left and right muscles were classified equally. Considering the anatomical structure, the boundary was defined as transverse line that encounter posterior pole of vertebral body so that the flexor muscle was mainly included in the anterior part and the extensor muscle was mainly included in the posterior part. Second, to analyze how HNP contributes to muscular atrophy, the muscles that make up the spinal column and innervated from lumbar spinal origin were defined as paraspinal muscles. And the parts of the whole trunk musculature excluding the paraspinal muscles were defined as peripheral muscles. After obtaining muscular portion of their image, we measure 2D surface area using function of CTAn to perform statistical analysis. APCT, Abdomen pelvis compute tomography.

Two factors were considered to analyze the correlation between HNP and skeletal muscle. First, we examined this relationship in terms of skeletal muscle function. The anterior muscles were composed of lumbar flexors, posterior muscles were composed of lumbar extensors as previously described (20), and left and right muscles were classified equally. Considering the anatomical structure, the boundary was defined as a transverse line that encounters the posterior pole of the vertebral body so that the flexor muscle was mainly included in the anterior part and the extensor muscle was mainly included in the posterior part. Second, to analyze how HNP contributes to muscular atrophy, the muscles that make up the spinal column and are innervated from lumbar spinal origin were defined as paraspinal muscles. Furthermore, the parts of the whole trunk musculature, excluding the paraspinal muscles, were defined as peripheral muscles. The paraspinal muscles consisted of the psoas, multifidus, and erector spinae muscles, whereas the peripheral muscles were defined as muscles excluding the paraspinal muscles, mainly consisting of the abdominal muscles. The left and right muscles were divided based on the line that bisects the vertebral body and the spinous process. After obtaining the muscular portion of their image, we measured the 2D surface area using the function of CTAn to perform statistical analysis (Figure 2).

Additionally, we analyzed the status of low skeletal muscle mass closely related to sarcopenia or obesity in both groups. First, sarcopenic status was determined by APCT for each patient. In this study, SMA was measured using CT to investigate whether the groups included met the diagnostic criteria for sarcopenia. For the cut-off value to define sarcopenia, we followed the criteria reported by Kim et al. in South Korea (21). They analyzed the SMA including all muscles on the selected axial images of the L3 vertebrae level with 11,845 participants (7,314 men and 4,531 women) who underwent abdominal CT scans. They suggested the cut-off points of SMA/BMI as 4.97 and 3.46 in men and women, respectively for the diagnosis of sarcopenia. Obesity was defined as a BMI ≥ 25.0 kg/m2 in adults, in accordance with the Asia-Pacific criteria of the World Health Organization guidelines (22).

### 2.3. Statistical analyses

We applied the propensity score matching (PSM) method for age and sex to reduce possible bias originating from the difference in patients’ demographic characteristics. Patients with HNP were matched with the healthy control group (non-HNP) based on a greedy algorithm of nearest neighbor matching at a 1:1 fixed ratio. HNP was considered the dependent variable, whereas baseline characteristics such as age, sex, height, weight, and BMI were considered predefined covariates. We obtained the same number of individuals for the HNP and non-HNP (Figure 1). Overall, 122 patients with HNP and 122 controls were enrolled before statistical comparison. The distribution of propensity scores and change of absolute standardized differences after propensity score matching were also investigated.

Pearson’s Chi-square test for categorical variables and student t-test for continuous variables were used to compare groups. A value of *p* < 0.05 was considered statistically significant. Continuous, normally distributed data are presented as mean ± standard deviation. The data manipulation and statistical analyses were performed using the R software, version 3.3.3 (The R Foundation for Statistical Computing, Vienna, Austria; http://www.R-project.org/).

## 3. Results

### 3.1. Demographics

Initial data showed demographic differences between the two groups for age, sex, and height. After propensity score matching was performed, no statistically significant difference between the two groups in age, sex, height, weight, and BMI was observed (*p*-value > 0.05 for all variables; Table 1; Supplementary Figures 1, 2). Moreover, after reclassification of individuals into men and women, no statistical difference was observed in the above characteristics (Supplementary Table 2). As expected, men had higher SMA values than women. The older the age, the smaller the SMA, and the larger the BMI, the larger the SMA.

**Table 1:** Demographics before and after PSM for age, sex, height, weight, and BMI.

	Before propensity score matching		*p*-value	After propensity score matching		*p*-value
	HNP	Non-HNP		HNP	Non-HNP	
	*n* = 134	*n* = 135		*n* = 122	*n* = 122	
Age (years old)	51.87 ± 14.33	48.71 ± 11.48	0.047^*^	51.11 ± 14.34	49.79 ± 11.23	0.424^*^
Sex			0.008^†^			0.245^†^
Men	70 (52.24%)	48 (35.56%)		64 (52.46%)	74 (60.66%)	
Women	64 (47.76%)	87 (64.44%)		58 (47.54%)	48 (39.34%)	
Height (m)	1.64 ± 0.10	1.68 ± 0.08	<0.001^*^	1.65 ± 0.09	1.67 ± 0.08	0.076^*^
Weight (kg)	66.19 ± 14.49	69.24 ± 12.07	0.062^*^	67.19 ± 14.65	68.09 ± 11.11	0.588^*^
BMI (kg/m^2^)	24.52 ± 4.02	24.43 ± 3.11	0.883^*^	24.40 ± 4.05	24.22 ± 2.90	0.694^*^

HNP, Herniated nucleus pulposus; BMI, Body mass index; PSM, Propensity score matching; ^*^T-test, ^†^Pearson’s Chi-square test.

### 3.2. Comparison of SMA by direction

Regarding the absolute value of SMA, no significant difference in total SMA was observed between patients with or without HNP (HNP 11629.52 ± 3607.80 mm^2^ vs. non-HNP 11667.70 ± 2679.11 mm^2^ *p*-value = 0.925). However, the HNP group had a significantly higher anterior SMA in both men and women than the non-HNP group (*p*-value = 0.008 for men and 0.048 for women; Table 2).

**Table 2:** Comparison of SMA by direction.

	SMA (mm^2^)					SMA ratio		
	Total	Anterior	Posterior	Right	Left	Anterior	Right	Left
**Total**								
HNP (*n* = 122)	11629.52 ± 3607.80	4770.75 ± 1966.95	6858.76 ± 1990.61	5779.60 ± 1857.17	5849.91 ± 1780.29	**0.40 ± 0.07**	0.50 ± 0.02	0.50 ± 0.02
Non-HNP (*n* = 122)	11667.70 ± 2679.11	4359.03 ± 1232.09	7308.67 ± 1602.17	5758.18 ± 1368.08	5909.53 ± 1338.69	**0.37 ± 0.04**	0.49 ± 0.02	0.51 ± 0.02
*p*-value	0.925	0.051	0.053	0.918	0.768	**<0.001**	0.246	0.246
**Men**								
HNP (*n* = 64)	13728.79 ± 3232.98	5751.19 ± 1760.77	7977.60 ± 1888.04	6871.44 ± 1646.94	6857.35 ± 1630.39	**0.42 ± 0.06**	0.50 ± 0.02	0.50 ± 0.02
Non-HNP (*n* = 74)	13383.35 ± 1849.43	5089.80 ± 942.35	8293.55 ± 1154.77	6623.53 ± 970.16	6759.82 ± 923.63	**0.38 ± 0.04**	0.49 ± 0.02	0.51 ± 0.02
*p*-value	0.452	**0.008**	0.247	0.293	0.673	**<0.001**	0.056	0.056
**Women**								
HNP (*n* = 58)	9313.07 ± 2381.25	3688.88 ± 1586.18	5624.19 ± 1230.96	4574.82 ± 1228.12	4738.26 ± 1180.28	**0.39 ± 0.08**	0.49 ± 0.02	0.51 ± 0.02
Non-HNP (*n* = 48)	9022.75 ± 1177.76	3232.43 ± 625.99	5790.31 ± 809.77	4424.09 ± 599.95	4598.66 ± 628.82	**0.36 ± 0.04**	0.49 ± 0.02	0.51 ± 0.02
*p*-value	0.417	**0.048**	0.407	0.413	0.439	**0.009**	0.994	0.994

SMA, Skeletal muscle area; HNP, Herniated nucleus pulposus; Ratio of anterior, Anterior SMA/Total SMA; Ratio of right, Right SMA/Total SMA. The student *t*-test was used for comparison between groups. Statistically significant when *p* < 0.05, indicated in bold.

Regarding the ratio of SMA, the ratio of anterior SMA was calculated by dividing the anterior SMA value by the total SMA value. In the non-HNP group, the ratio of anterior SMA was 0.37 ± 0.04. However, the ratio of anterior SMA was significantly higher at 0.40 ± 0.07 in the HNP group (*p*-value < 0.001). Specifically, in both men and women, the ratio of anterior SMA was higher with statistical significance in the HNP group compared to that in the non-HNP group (men: HNP 0.42 ± 0.06 vs. non-HNP 0.38 ± 0.04, *p*-value < 0.001, women: HNP 0.39 ± 0.08 vs. non-HNP 0.36 ± 0.04, *p*-value = 0.009). Next, the ratio of left and right SMA was also analyzed. However, no significant difference was observed regarding sex between the HNP and non-HNP groups (Table 2).

### 3.3. Comparison between paraspinal and peripheral SMA

After comparing the paraspinal and peripheral SMA, no significant difference between left and right. Therefore, the absolute paraspinal SMA value was irrelevant to the HNP or non-HNP groups. However, regarding peripheral muscles, statistical significance was observed in the left and right imbalance in men HNP patients (right peripheral SMA: HNP men 2643.25 ± 846.57 vs. non-HNP men 2392.81 ± 477.45 mm^2^, *p*-value = 0.039). Furthermore, after comparing the ratio of the peripheral SMA by total SMA, HNP patients had a higher peripheral SMA ratio than the non-HNP with statistical significance in total (ratio of both side peripheral: HNP 0.62 ± 0.19 vs. non-HNP 0.57 ± 0.08, *p*-value = 0.014, Ratio of right peripheral: HNP 0.62 ± 0.19 vs. non-HNP men 0.56 ± 0.10, *p*-value = 0.006). In addition, it was found that in men with HNP, the ratio of peripheral muscle to the right was larger than that of non-HNP (Ratio of right peripheral: HNP men 0.63 ± 0.15 vs. non-HNP men 0.57 ± 0.09, *p*-value = 0.004; Table 3).

**Table 3:** Comparison between paraspinal and peripheral SMA.

	SMA (mm^2^)					SMA (ratio of peripheral)		
	Total	Paraspinal		Peripheral		Both side	One side	
	Paraspinal + peripheral	Right	Left	Right	Left	Right + Left	Right	Left
**Total**								
HNP (*n* = 122)	11629.52 ± 3607.80	3576.02 ± 1059.93	3633.73 ± 1062.66	2203.58 ± 958.74	2216.19 ± 929.98	**0.62 ± 0.19**	**0.62 ± 0.19**	0.62 ± 0.22
Non-HNP (*n* = 122)	11667.70 ± 2679.11	3686.89 ± 851.89	3752.47 ± 849.35	2071.28 ± 586.15	2157.06 ± 563.48	**0.57 ± 0.08**	**0.56 ± 0.10**	0.58 ± 0.09
*p*-value	0.925	0.369	0.336	0.195	0.549	**0.014**	**0.006**	0.062
**Men**								
HNP (*n* = 64)	13728.79 ± 3232.98	4228.19 ± 975.78	4272.40 ± 1015.72	2643.25 ± 846.57	2584.95 ± 872.68	**0.62 ± 0.16**	**0.63 ± 0.15**	0.62 ± 0.21
Non-HNP (*n* = 74)	13383.35 ± 1849.43	4230.72 ± 596.53	4301.67 ± 577.98	2392.81 ± 477.45	2458.15 ± 458.60	**0.57 ± 0.08**	**0.57 ± 0.09**	0.57 ± 0.09
*p*-value	0.452	0.986	0.839	**0.039**	0.299	**0.019**	**0.004**	0.111
**Women**								
HNP (*n* = 58)	9313.07 ± 2381.25	2856.38 ± 572.68	2928.99 ± 541.77	1718.43 ± 838.19	1809.27 ± 820.38	0.61 ± 0.22	0.60 ± 0.23	0.62 ± 0.22
Non-HNP (*n* = 48)	9022.75 ± 1177.76	2848.49 ± 363.45	2905.78 ± 362.36	1575.60 ± 341.67	1692.88 ± 357.76	0.57 ± 0.09	0.56 ± 0.11	0.58 ± 0.10
*p*-value	0.417	0.932	0.793	0.240	0.333	0.217	0.176	0.327

SMA, Skeletal muscle area; HNP, Herniated nucleus pulposus; Ratio of peripheral, Peripheral SMA/Total SMA. The student *t*-test was used for comparison between groups. Statistically significant when *p* < 0.05, indicated in bold.

### 3.4. Skeletal muscle mass comparison according to criteria for diagnosis of sarcopenia between the HNP and Non-HNP groups

Regarding the analysis of skeletal muscle mass according to the criteria for sarcopenia diagnosis, no statistical difference in the prevalence of low skeletal muscle mass (sarcopenia) was observed between the groups of HNP and non-HNP (HNP 28.7% vs. non-HNP 24.6%% *p*-value = 0.562; Supplementary Table 3). Moreover, no statistical significance in low skeletal muscle mass with obesity was observed (HNP 57.1% vs. non-HNP 56.7%, p-value = 1.000; Supplementary Table 4).

## 4. Discussion

In this retrospective cross-sectional study that performed APCT with propensity score matching analysis, we found that the ratio of anterior muscle SMA was greater in HNP group than in the non-HNP group. In addition, it was found that HNP patients had a significant higher peripheral SMA ratio than that non-HNP patients. Moreover, peripheral SMA was more enlarged unilaterally in HNP. Contrary to the results of SMA, low skeletal muscle mass closely related to sarcopenia with or without obesity was not associated with the HNP.

Compared to other studies, the strength of this study is that the results were analyzed by comparing them with the control group (those without HNP). The trend of previous studies was that only data on patients with HNP were collected (17, 18, 23–26). However, in this study, data about whole abdominal muscle around the spine could be collected even in individuals without HNP using APCT. Moreover, APCT can measure not only the back muscles but also the muscles of the abdomen; hence, the overall muscle balance can be measured. In addition, the impact of other variables on the outcome was reduced through PSM.

There are several studies on using CT to objectively quantify and measure muscle volume (27, 28). However, for research purposes, such measurements are often collected retrospectively and opportunistically; extracting measurements from CT scans obtained during the normal course of clinical care adds no additional risk and is convenient (28). In addition, in this study, we analyzed not only the paraspinal muscles but also all trunk muscles, including the abdominal muscles using APCT. Compared to lumbar spine MRI or CT, APCT has a wider imaging range. In addition, only the muscles were analyzed using the quantification program, selectively excluding fat, ligaments, and vessels.

Compared to MRI, APCT scan has the disadvantage of radiation exposure, but several special advantages exist. First, it takes a short time to test. Secondly, it is cheaper, and thirdly, when studying muscles, spine MRI has a narrow range of images that can be included in an axial image, so only the spine extensor can be measured. On the other hand, if APCT is used, the whole trunk muscle can be included in the axial cut at once, making it easy to measure SMA. In this aspect, the authors believe a APCT scan is useful for studying low skeletal muscle or muscle imbalance.

In the study reported by Kim et al. (17), multifidus showed a decrease in SMA on the affected HNP side and no statistically significant decrease in psoas muscle when symptom duration was 3 months or more. This study concluded by comparing the normal and unilateral regions where HNP occurred in patients with HNP. However, in our study, the balance of all trunk muscles was compared with that of non-HNP individuals. Patients with HNP showed peripheral muscle imbalance compared to non-HNP individuals. In addition, even in the presence of HNP, no difference between the left and right paraspinal muscle SMA was observed (Supplementary Table 5). Therefore, it is unlikely that HNP will cause muscle atrophy if the duration of HNP is not long.

Several opinions have been suggested as factors that cause paraspinal asymmetry in men. Stewart et al. (29) have reported significant trunk muscle asymmetry in elite athletes performing asymmetrical sports. Fortin et al. (26) suggested that people with more physically demanding jobs or greater exercise and sports participation may have less asymmetry in paraspinal muscle size and fatty infiltration. According to a prospective cohort study by Linek et al. (30), as a result of the follow-up of 97 adolescent male soccer players without LBP in the beginning, LBP occurred 6 months later, and a statistically significant asymmetry of obliquus internus occurred (odds ratio 2.4). Similarly, an imbalance of oblique internus has been reported in physically active adolescent girls (31). According to a study by Iwai (32) that measured muscle SMA using MRI, a significant imbalance between the left. Right sides of the trunk muscle in collegiate men combat sports athletes with lumbar intervertebral disc degeneration was observed [abdominis obliques left 44.7 ± 8.7 (42.6–46.8) vs. right 43.8 ± 8.1 (41.8–45.7) *p* = 0.040, quadratus lumborum left 23.3 ± 17.7 (19.1–27.6) vs. right 21.8 ± 17.2 (17.7–25.9) *p* < 0.001]. According to a report by Malliaropoulos et al. (33), in a 20-year observational cohort study with 130 Elite Track and Field Athletes, 46.9% of LBP was discogenic. In our study, the SMA and ratio of the right peripheral muscle of a male HNP patient was significantly higher than that of non-HNP individuals. From this study results, the asymmetrical enlargement of the peripheral muscles in our study suggests the possibility that asymmetric overactivity of the body causes asymmetric hypertrophy of the peripheral muscles. Since the paraspinal muscle is symmetrical, it can be considered that the progression of degenerative changes in HNP due to the asymmetric use of the trunk muscle is more likely to occur than atrophy of the muscle due to nerve compression caused by HNP. Considering the results of the above-mentioned studies, LBP may be associated with atrophy of paraspinal muscle in older adults with advanced disc degeneration. However, in the case of relatively young patients, hypertrophic changes in the muscle may be caused by exercise pattern, contributing to muscle imbalance. In our study, HNP patients did not show muscle atrophy compared to normal individuals and showed unilateral muscle hypertrophy. Moreover, the average age of participants in this study was approximately 50 years old, and compared to the elderly, they are considered young and active. Compared to the previous literature, it is worth reconsidering the relationship between spinal muscle muscular atrophy and HNP in young patients with HNP.

According to the Asian Working Group for Sarcopenia 2019, sarcopenia was defined as low appendicular skeletal muscle mass plus low muscle strength or low physical performance. Severe sarcopenia was defined as low appendicular skeletal muscle mass plus low muscle strength and low physical performance (34). In accordance with our results, according to a study by Fujita et al. using MRI and skeletal muscle index, there is no relationship between intervertebral disc degeneration and sarcopenia (35). Park et al. (36) reported that sarcopenia was more prevalent in patients with lumbar spinal stenosis compared with matched controls and concluded that the impacts of sarcopenia on disability, quality of life, and physical performance were more pronounced in patients with lumbar spinal stenosis. Furthermore, physical inactivity due to spinal disorders may cause an accelerated decline in appendicular and trunk skeletal muscle mass, muscle strength, and functional capacity compared with controls (37). However, only few studies have investigated the relationship between sarcopenia and intervertebral disc degeneration; hence, the relationship is unclear. In our study, which analyzed the relationship with sarcopenia in the HNP group, no statistically significant correlation was observed. The results of this study may be related to the duration of HNP disease. Considering the results of this study and that of previous studies, the relationship between HNP and sarcopenia seems unlikely. However, it is believed that sarcopenia may occur when HNP progresses for a long time and develops into lumbar spinal stenosis.

As a limitation of this study, this study is a retrospective cross-sectional study, and the relationship between cause and effect with HNP and muscle imbalance was unclear. However, considering that the flexor muscles were larger in patients with HNP, the imbalanced use of muscles may cause HNP. A prospective longitude study is further needed to identify the causes and effects related to this. Additionally, the direction in which the disc protruded and the severity of HNP were not investigated in this study. Therefore the direction and severity of muscle atrophy could not be matched with the specific character of the patient’s HNP. To evaluate sarcopenic status, only SMA was measured from APCT. There were no data on handgrip strength or physical performance; therefore, this study did not meet the sarcopenia criteria corresponding to the AWGS 2019. In addition, in this paper, it was impossible to analyze the patient’s underlying disease (e.g., diabetes and cardiovascular diseases) or physical performance due to insufficient medical records due to a retrospective literature review. Since these matters may affect the analysis of the results, controlling these variables in future studies is recommended.

## 5. Conclusion

According to several previous publications, core muscle imbalance not only affects LBP but also significantly impacts overall health and quality of life. In this study using APCT, the lumbar flexor muscle was larger in patients with HNP than in healthy individuals. For this reason, it may be good to plan future exercise training to strengthen the lumbar extensor. Therefore, our study showed trunk muscle imbalance in patients with HNP. Moreover, it is necessary to establish a treatment and rehabilitation plan for correcting core muscle imbalance through continuous follow-up studies. APCT can be useful for planning studies related to muscle imbalance and evaluating the results.

## Data availability statement

The original contributions presented in the study are included in the article/supplementary material, further inquiries can be directed to the corresponding author.

## Ethics statement

This study was approved by the CHA Bundang Medical Center Institutional Review Board (number 2021-06-037). Written informed consent for participation was not required for this study in accordance with the national legislation and the institutional requirements.

## Author contributions

C-HO: project administration, data curation, formal analysis, and writing the original draft. D-ES: critic review and supervision. SY: data curation, formal analysis, and writing the original draft. JO: data curation and formal analysis. YL: data curation. SL: conceptualization, funding acquisition, investigation, methodology, and manuscript review and editing. All authors contributed to the article and approved the submitted version.

## Funding

This work was supported by the National Research Foundation of Korea (NRF) grant funded by the Korea government (MSIT) (Nos. NRF-2021R1A4A3023587, 2022R1A2C2005916).

## Conflict of interest

The authors declare that the research was conducted in the absence of any commercial or financial relationships that could be construed as a potential conflict of interest.

## Publisher’s note

All claims expressed in this article are solely those of the authors and do not necessarily represent those of their affiliated organizations, or those of the publisher, the editors and the reviewers. Any product that may be evaluated in this article, or claim that may be made by its manufacturer, is not guaranteed or endorsed by the publisher.
