# Supplementary material for: Comparison of whole trunk muscle mass between healthy and lumbar herniated nucleus pulposus patients using abdominal pelvic computed tomography
Source: Front Med (Lausanne). 2023 Aug 1;10:1190021. doi: 10.3389/fmed.2023.1190021 (PMC10428013; doi:10.3389/fmed.2023.1190021)
Supplement: Supplementary file 1 [file Data_Sheet_1.docx]

**Supplemental Table 1. Purpose of APCT evaluation of recruited HNP patients**

| **Symptom** | **Men**  **n = 70** | **Women**  **n = 64** |
| --- | --- | --- |
| **Abdominal pain** |  |  |
| **Acute gastroenteritis** | 4 | 5 |
| **Acute colitis** | 5 | 4 |
| **Appendicitis** | 5 | 8 |
| **Ureteric stone** | 5 | 3 |
| **Gynecologic disease** | 0 | 5 |
| **No Significant Findings** | 12 | 8 |
| **Trauma** |  |  |
| **Liver contusion** | 23 | 2 |
| **No Significant findings** | 12 | 10 |
| **Hematochezia/melena** |  |  |
| **Peptic ulcers** | 1 | 3 |
| **Diverticulitis** | 3 | 2 |
| **Gastritis** | 1 | 0 |
| **Crohn’s disease** | 1 | 0 |
| **Others** | 4 | 4 |
| **Others** |  |  |
| **Chronic kidney disease** | 7 | 5 |
| **Jaundice** | 2 | 1 |
| **Others** | 5 | 4 |

APCT: Abdomen pelvis computed tomography, HNP: Herniated nucleus pulposus

**Supplemental Table 2. Demographics for HNP and non-HNP by sex after PSM**

|  | **Men** | | **P-value** | **Women** | | **P-value** |
| --- | --- | --- | --- | --- | --- | --- |
|  | **HNP**  **n = 64** | **Non-HNP**  **n = 74** |  | **HNP**  **n = 58** | **Non-HNP**  **n = 48** |  |
| **Age (years old)** | 50.00 ± 14.92 | 49.22 ± 10.30 | 0.724 | 52.33 ± 13.6 | 50.67 ± 12.59 | 0.521 |
| **Height (m)** | 1.71 ± 0.07 | 1.72 ± 0.06 | 0.152 | 1.59 ± 0.06 | 1.59 ± 0.05 | 0.879 |
| **Weight (kg)** | 73.35 ± 14.42 | 73.39 ± 8.55 | 0.986 | 59.94 ± 9.62 | 59.94 ± 9.62 | 0.828 |
| **BMI (kg/m^2^)** | 25.03 ± 4.32 | 24.66 ± 2.41 | 0.541 | 23.54 ± 3.45 | 23.54 ± 3.45 | 0.825 |

In subgroup analysis there was no statistically significant difference between the two groups in age, height, weight, and BMI. HNP: Herniated nucleus pulposus, PSM: Propensity score matching, BMI: Body mass index; The student t-test was used for comparison between groups.

**Supplemental Table 3. Skeletal muscle mass comparison according to diagnosis sarcopenia criteria between HNP and Non-HNP**

| **Prevalence** | **HNP**  **n = 122** | **Non-HNP**  **n = 122** | **P-value** |
| --- | --- | --- | --- |
| **Low skeletal muscle mass** |  |  | 0.562 |
| **Yes** | 35 (28.7%) | 30 (24.6%) |  |
| **No** | 87 (71.3%) | 92 (75.4%) |  |

HNP: Herniated nucleus pulposus, BMI: Body mass index, SMA: Skeletal muscle area; The Pearson’s chi-square test was used for comparison between groups

**Supplemental Table 4. BMI comparison between Low skeletal muscle mass and Normal skeletal muscle mass in both HNP and non-HNP group**

| **BMI (kg/m^2^)** | **HNP** | **Non-HNP** | **P-value** |
| --- | --- | --- | --- |
| **Low skeletal muscle mass** |  |  | 1.000 |
| **BMI ≥ 25** | 20 (57.1%) | 17 (56.7%) |  |
| **BMI < 25** | 15 (42.9%) | 13 (43.3%) |  |
| **Normal skeletal muscle mass** |  |  | 0.437 |
| **BMI ≥ 25** | 21 (24.1%) | 28 (30.4%) |  |
| **BMI < 25** | 66 (75.9%) | 64 (69.6%) |  |

HNP: Herniated nucleus pulposus, BMI: Body mass index; The Pearson’s chi-square test was used for comparison between groups

**Supplemental Table 5. Comparison of SMA according to the side of paraspinal muscle**

|  | **Right Paraspinal SMA (mm^2^)** | **Left Paraspinal SMA (mm^2^)** | **P-value** |
| --- | --- | --- | --- |
| **Men** |  |  |  |
| **HNP (n = 64)** | 4228.19 ± 975.78 | 4272.40 ± 1015.72 | 0.802 |
| **Non-HNP (n = 74)** | 4230.72 ± 596.53 | 4301.67 ± 577.98 | 0.464 |
| **P-value** | 0.986 | 0.839 |  |
| **Women** |  |  |  |
| **HNP (n = 58)** | 2856.38 ± 572.68 | 2928.99 ± 541.77 | 0.484 |
| **Non-HNP (n = 48)** | 2848.49 ± 363.45 | 2905.78 ± 362.36 | 0.441 |
| **P-value** | 0.932 | 0.793 |  |
| **Total** |  |  |  |
| **HNP (n = 122)** | 3576.02 ± 1059.93 | 3633.73 ± 1062.66 | 0.671 |
| **Non-HNP (n = 122)** | 3686.89 ± 851.89 | 3752.47 ± 849.35 | 0.548 |
| **P-value** | 0.369 | 0.336 |  |

SMA: Skeletal muscle area, HNP: Herniated nucleus pulposus; The student t-test was used for comparison between groups.

**Supplemental figure 1. Distribution of propensity scores before and after matching**


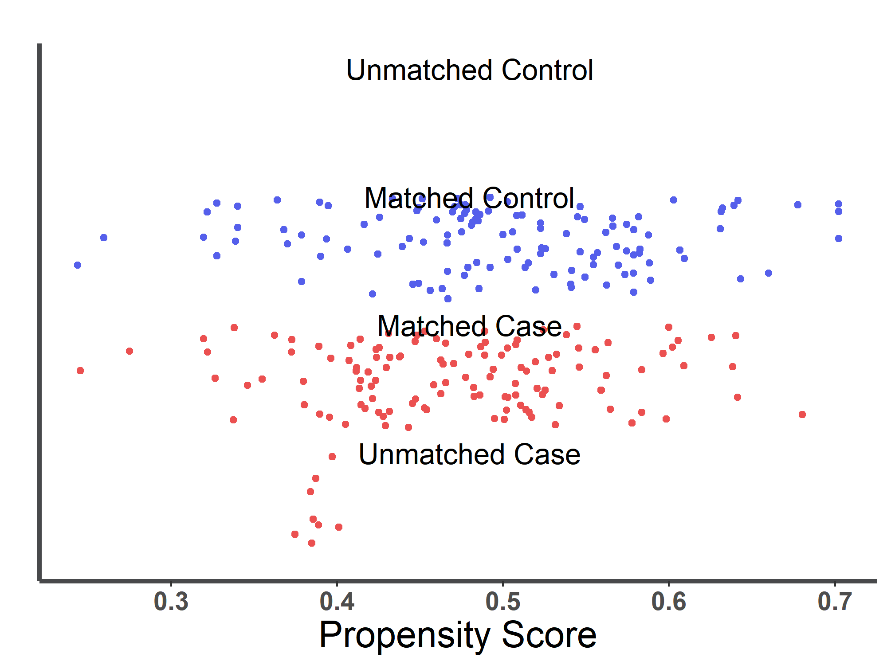


**Supplemental figure 2. Change of absolute standardized differences before and after propensity score matching**

**
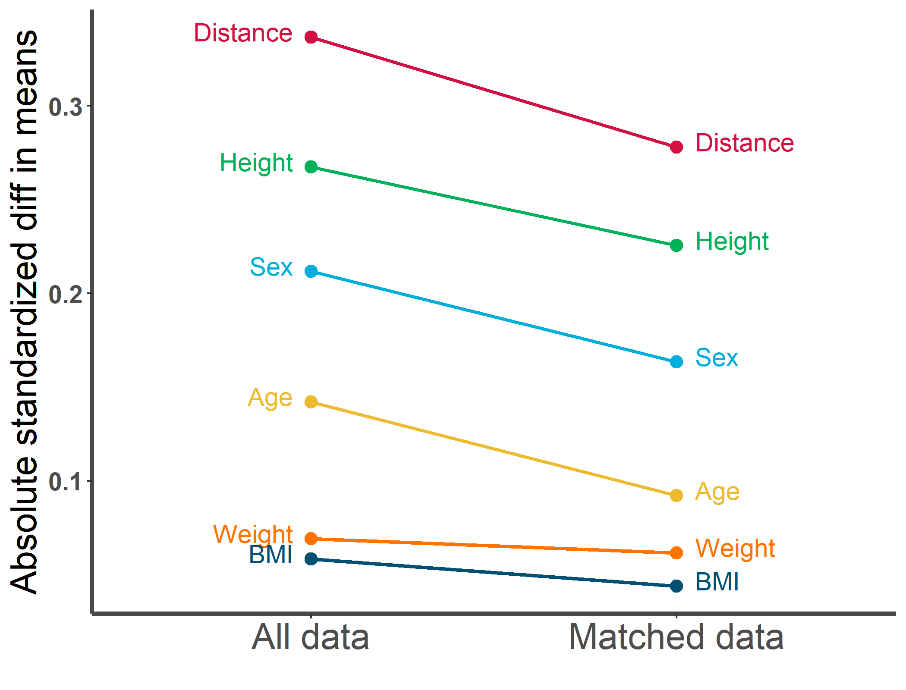
**
